# Cytosine deaminase as a negative selectable marker for the microalgal chloroplast: a strategy for the isolation of nuclear mutations that affect chloroplast gene expression

**DOI:** 10.1111/tpj.12675

**Published:** 2014-09-18

**Authors:** Rosanna E B Young, Saul Purton

**Affiliations:** Algal Research Group, Institute of Structural and Molecular Biology, University College LondonGower Street, London, WC1E 6BT, UK

**Keywords:** *Chlamydomonas reinhardtii*, microalgae, chloroplast, post-transcriptional regulation, cytosine deaminase, 5-fluorocytosine, 5-fluorouracil, technical advance

## Abstract

Negative selectable markers are useful tools for forward-genetic screens aimed at identifying *trans*-acting factors that are required for expression of specific genes. Transgenic lines harbouring the marker fused to a gene element, such as a promoter, may be mutagenized to isolate loss-of-function mutants able to survive under selection. Such a strategy allows the molecular dissection of factors that are essential for expression of the gene. Expression of individual chloroplast genes in plants and algae typically requires one or more nuclear-encoded factors that act at the post-transcriptional level, often through interaction with the 5′ UTR of the mRNA. To study such nuclear control further, we have developed the *Escherichia coli* cytosine deaminase gene *codA* as a conditional negative selectable marker for use in the model green alga *Chlamydomonas reinhardtii*. We show that a codon-optimized variant of *codA* with three amino acid substitutions confers sensitivity to 5-fluorocytosine (5-FC) when expressed in the chloroplast under the control of endogenous promoter/5′ UTR elements from the photosynthetic genes *psaA* or *petA*. UV mutagenesis of the *psaA* transgenic line allowed recovery of 5-FC-resistant, photosynthetically deficient lines harbouring mutations in the nuclear gene for the factor TAA1 that is required for *psaA* translation. Similarly, the *petA* line was used to isolate mutants of the *petA* mRNA stability factor MCA1 and the translation factor TCA1. The *codA* marker may be used to identify critical residues in known nuclear factors and to aid the discovery of additional factors required for expression of chloroplast genes.

## Introduction

The coordination of nuclear and chloroplast gene expression in both microalgae and higher plants is largely effected through nuclear-encoded proteins targeted to chloroplasts. These *trans*-acting factors each regulate the expression of one or a few specific chloroplast genes, such as those encoding subunits of the photosynthetic complexes photosystem I (PSI), photosystem II (PSII), the cytochrome *b*_6_*f* complex (cyt *b*_6_*f*), ATP synthase and Rubisco. Such factors act mainly at the post-transcriptional level by binding to chloroplast transcripts, often through the 5′ UTR (Marín-Navarro *et al.*, 2007; Bohne *et al.*, 2009). These factors have been termed ‘nuclear-encoded regulators of organellar gene expression’ (ROGEs), and have been characterized in both higher plants and the unicellular green alga *Chlamydomonas reinhardtii* (Woodson and Chory, 2008). Examples from the latter include NAC2, which stabilizes the *psbD* mRNA encoding the core PSII subunit D2 and has been used to create a *psbD* 5′ UTR-linked system for inducible gene expression in the chloroplast (Nickelsen *et al.*, 1999; Boudreau *et al.*, 2000; Rochaix *et al.*, 2014), MCA1, which is required for the stabilization of *petA* mRNA encoding the apo-cytochrome *f* subunit of cyt *b*_6_*f* and plays a central role in the regulation of cyt *b*_6_*f* biogenesis (Loiselay *et al.*, 2008), and TCA1, which is required for translation of the same transcript (Wostrikoff *et al.*, 2001; Raynaud *et al.*, 2007). Most ROGEs interact with their cognate RNA via multiple tandem repeats that form pairs of RNA-binding α-helices (Hammani *et al.*, 2014), with different classes of such binding proteins being defined based on the length of each degenerate repeat, i.e. tetratricopeptide repeat proteins with 34 amino acid repeats, pentatricopeptide repeat (PPR) proteins with 35 amino acid repeats, and octotricopeptide repeat proteins with 38 amino acid repeats. As an example, MCA1 is a PPR protein that is found throughout the Chlorophyta (Tourasse *et al.*, 2013), and in *C. reinhardtii* has been shown to interact with the first 21 bases of the *petA* transcript, thereby protecting it from 5′→3′ degradation (Loiselay *et al.*, 2008).

Both chloroplast and nuclear genetic engineering are well established in *C. reinhardtii* (Purton, 2007; Gimpel *et al.*, 2013), and as such this alga is ideally suited for molecular genetic studies of ROGEs. However, such studies in *C. reinhardtii* and other microalgae would be advanced by the availability of a conditional negative selectable marker for the chloroplast. Placing the marker downstream of the 5′ UTR of a chloroplast gene of interest, mutagenizing the cell population and selecting for cell lines that no longer express the marker would allow isolation of mutants that are specifically affected in ROGEs that interact with that particular 5′ UTR element. This method would be more efficient than traditional screening of large numbers of random mutant colonies for a small subset that are affected in the expression of particular chloroplast genes (Gumpel *et al.*, 1995), which is time-consuming and generally involves use of a series of phenotypic assays such as altered chlorophyll fluorescence, pulse labelling of chloroplast proteins and Northern blotting to identify rare nuclear mutants affected in the stability or translation of a specific chloroplast transcript.

The *codA* gene of *Escherichia coli* encodes cytosine deaminase, and has previously been used as a negative selectable marker in a range of organisms that naturally lack this enzyme activity, including mammalian cell lines (Mullen *et al.*, 1992), the nucleus and plastids of higher plants (Stougaard, 1993; Kobayashi *et al.*, 1995; Serino and Maliga, 1997; Dai *et al.*, 2001), and actinobacteria (Dubeau *et al.*, 2009). The enzyme catalyses conversion of cytosine to uracil and ammonia for pyrimidine salvage, but can also convert the synthetic compound 5-fluorocytosine (5-FC) to the toxic product 5-fluorouracil (5-FU). 5-FU can be metabolized into 5-fluorodeoxyuridine monophosphate, which inhibits thymidylate synthetase and therefore DNA synthesis, or into 5-FU triphosphate, whose incorporation into RNA leads to inhibition of protein synthesis (Vermes *et al.*, 2000). Expression of the marker therefore gives rise to a 5-FC-sensitive phenotype, and, in a clinical setting, a combination of 5-FC with *codA* gene therapy may be used for the targeted treatment of tumours (Zarogoulidis *et al.*, 2013). As a consequence, the structure and function of the 300 kDa CodA hexamer have been intensively studied (Ireton *et al.*, 2002; Fuchita *et al.*, 2009; Hall *et al.*, 2011).

Here we describe the development of a modified cytosine deaminase gene as a easy-to-use negative selectable marker for the *C. reinhardtii* chloroplast, and demonstrate its utility in the isolation of novel mutant alleles of three previously identified ROGEs that are required for expression of *psaA* (the translation factor TAA1) or *petA* (the mRNA stability factor MCA1 and the translation factor TCA1).

## Results

### 5-FU, but not 5-FC, inhibits the growth of *C. reinhardtii*

Cytosine deaminase (CD) enzymes are found in most bacteria and yeast, but are absent in mammals, higher plants and actinobacteria. CD activities have not been reported in green algae, and similarity searches of the *C. reinhardtii* genome sequence did not find any predicted gene products with significant matches to CD proteins from either *E. coli* (accession number NP_414871) or *Saccharomyces cerevisiae* (accession number NP_015387).

**Figure 1:**
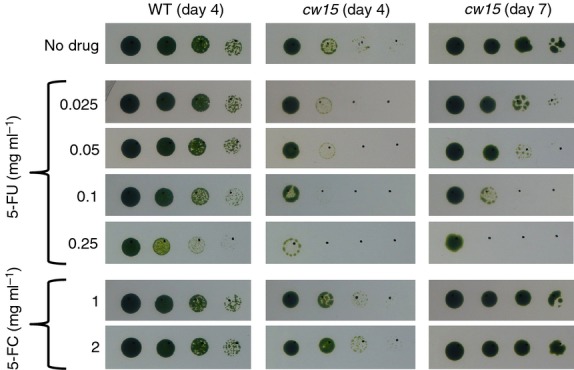
Wild-type and cell-wall mutant strains of *C. reinhardtii* are resistant to 5-fluorocytosine but sensitive to 5-fluorouracil. *C. reinhardtii* strains wild-type CC-621 (mt−) and *cw15*.J3 (mt−), labelled ‘WT’ and ‘*cw15*’, respectively, were adjusted to equal optical densities and spotted onto TAP agar containing 5-FU, 5-FC or no drug in serial fivefold dilutions (left to right). Plates were incubated under 50 μE m^−2^ sec^−1^ light.

For *codA* to function as a negative selectable marker in the algal chloroplast, the growth of wild-type cells must not be inhibited by exogenous 5-FC but must be inhibited by 5-FU. Figure1 demonstrates that these initial requirements are met: concentrations of up to 2 mg ml^−1^ 5-FC (the highest level tested) do not negatively affect growth, whilst 5-FU slows growth at concentrations as low as 25 μg ml^−1^ and severely affects growth at 250 μg ml^−1^. The latter results agree with those of Jacobson *et al*. (1964), who tested a range of compounds against *C. reinhardtii* and found that 130 μg ml^−1^ 5-FU affected cell division. Overall, our growth experiments suggest that there is no natural CD activity in *C. reinhardtii*. 5-FU has a greater effect on the cell wall-deficient mutant *cw15*.J3 than the walled wild-type strain (Figure1), suggesting that the cell wall acts as a partial barrier to 5-FU uptake. A cell wall-deficient mutant was used for the remainder of the work described here.

### An optimized version of *E. coli codA* expressed in the *C. reinhardtii* chloroplast confers 5-FC sensitivity

**Figure 2:**
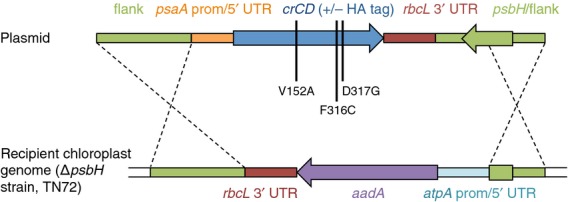
An optimized version (*crCD*) of the *E. coli* cytosine deaminase gene *codA* was introduced into the *C. reinhardtii* chloroplast under the control of the promoter and 5′ UTR from *psaA* exon 1. In addition to codon optimization of *codA*, three amino acid substitutions were made (vertical black lines). Dashed lines show the positions of homologous recombination.

**Figure 3:**
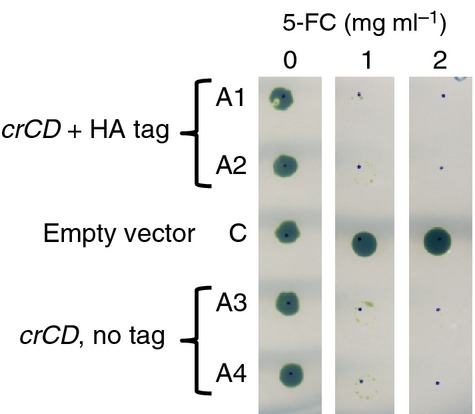
When expressed in the *C. reinhardtii* chloroplast, *crCD* confers sensitivity to 5-fluorocytosine. Four independent, homoplasmic *crCD* transformants (A1–A4) and a control cell line (C) transformed with empty vector were adjusted to equal optical densities and spotted onto TAP agar with or without 5-FC. Plates were incubated under 50 μE m^−2^ sec^−1^ light for 11 days.

**Figure 4:**
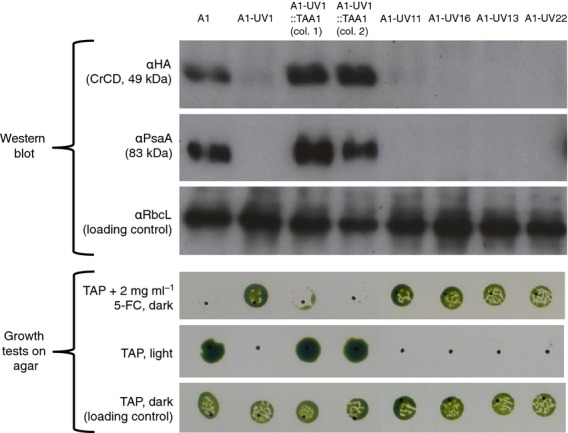
Western analysis and growth tests. UV treatment and plating on 5-FC allowed selection of mutants affected in expression from the *psaA* exon 1 promoter/5′ UTR. The original phenotype was restored by complementation with the *TAA1* gene. Cell lines were grown in liquid TAP medium in darkness. For Western analysis, equalized lysates were subjected to SDS–PAGE; three identical blots were probed with antibodies as indicated on the left. For growth tests, cells were spotted onto TAP agar with or without 5-FC. Plates were incubated in darkness or under 50 μE m^−2^ sec^−1^ light. Sensitivity to 5-FC (first row) correlates with CrCD expression, as expected. Light sensitivity (second row) is a sign of a photosystem I defect, in this case lack of PsaA protein, in the five UV mutants.

In initial chloroplast transformation experiments using native *codA* genes from *E. coli*, the cyanobacterium *Synechocystis* sp. PCC 6803 or the yeast *Saccharomyces cerevisiae*, linked to an *atpA* promoter/5′ UTR element, transgenic *C. reinhardtii* lines were produced for all three genes. However, no cyanobacterial or yeast CD was detected following immunoblotting with an antibody against the added haemagglutinin (HA) epitope tag, and only low levels were detected in the *C. reinhardtii* lines expressing *E. coli codA* (Figure S1a). Furthermore, the level of the *E. coli* CD was not high enough to allow negative selection on 5-FC (Figure S1b). To increase the expression level and activity in the chloroplast, two modifications were made. First, a multi-optimized version of the *E. coli codA* gene (named *crCD*) was synthesized. The synthetic *crCD* gene combines codon optimization for the *C. reinhardtii* chloroplast (increasing the *codA* codon adaptation index from 0.150 to 0.972) with the introduction of three amino acid substitutions that have previously been shown to increase the affinity of the enzyme for 5-FC as a substrate, both *in vitro* and in *E. coli* and cancer cell lines (Fuchita *et al.*, 2009). Second, the *atpA* promoter/5′ UTR element was replaced with that from *psaA* exon 1, as Michelet *et al*. (2011) reported higher levels of transgene expression with the *psaA* element. Transformation plasmids were constructed containing two versions of the *psaA::crCD* sequence, one in which the *crCD* coding region had been extended to add a C-terminal HA epitope tag to the CD protein for detection. As illustrated in Figure2, the transgene was targeted by homologous recombination to a neutral site within the *C. reinhardtii* chloroplast genome downstream of the PSII gene *psbH*. Selection for transformants involved restoration of a functional *psbH*, and hence phototrophy, in a PSII-deficient recipient line in which *psbH* had been deleted (Economou *et al.*, 2014). Transgenic lines (named A1–A4) were obtained for both constructs, with integration of the transgene and homoplasmicity of the genome in all the lines being confirmed by PCR analysis (Figure S2). All of the cell lines show highly reduced growth on 5-FC (Figure3), indicating that the recombinant CrCD protein is synthesized and active in the chloroplast, and that the presence of the HA tag has no effect on the activity of the enzyme. The HA-tagged version was used for all subsequent experiments. The synthesis of CrCD was further confirmed for the HA-tagged line A1, for which anti-HA antibodies detected a protein of the expected size for CrCD (49 kDa) in the cell extract, as shown in Figure4.

### UV treatment of *crCD* cell lines allows selection for *trans*-acting factor mutants

#### Isolation of taa1 mutants by linking crCD to the psaA exon 1 promoter and 5′ UTR

To investigate whether *crCD* may be used as a tool in forward-genetics screens for isolating specific classes of nuclear mutants, a UV mutagenesis screen with 5-FC selection was performed using transformant line A1. In this cell line, both *crCD* and exon 1 of the native *psaA* gene, encoding a core subunit of PSI, use the *psaA* exon 1 promoter and 5′ UTR. Any nuclear mutation that specifically reduces expression from this promoter or affects the stability or translation of transcripts containing this 5′ UTR will reduce or prevent synthesis of both PsaA and CrCD, resulting in a photosynthesis-deficient, 5-FC-resistant phenotype.

In an initial small-scale screen, A1 was UV-mutagenized and plated on medium containing acetate (to support heterotrophic growth of photosynthetic mutants) and 2 mg ml^−1^ 5-FC. The plates were incubated in the dark as PSI-deficient mutants are particularly light-sensitive (Spreitzer and Mets, 1981). One 5-FC-resistant colony, named A1-UV1, was obtained. It was shown to be deficient in the accumulation of CrCD and PsaA, and to grow poorly in the light (Figure4), suggesting impaired expression of genes that use the *psaA* exon 1 promoter/5′ UTR.

**Figure 5:**
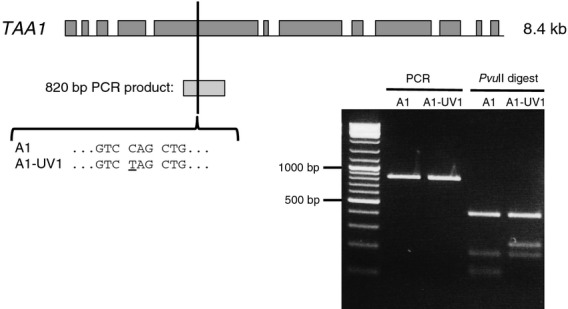
Identification of a Q633STOP mutation in the *TAA1* gene of UV mutant A1-UV1 by DNA sequencing, and confirmation by restriction digest. Dark grey boxes represent exons. The position of an 820 bp PCR product surrounding the premature stop codon is indicated by a pale grey box. This product was digested with *Pvu*II, which cuts at the sequence CAGCTG; mutation of this sequence causes a change in the restriction pattern relative to the parental A1 strain, as shown by agarose gel electrophoresis (right). The left lane contains GeneRuler DNA Ladder Mix (Thermo Scientific).

A nucleus-encoded ROGE that is thought to be required specifically for PsaA expression has recently been identified and named TAA1 (M. Goldschmidt-Clermont, Department of Botany and Plant Biology, University of Geneva, Switzerland; personal communication). To investigate whether the cell line A1-UV1 contained a mutation in *TAA1*, we transformed it with a plasmid (64B4E) containing a wild-type copy of the gene, and selected for the restoration of photosynthetic growth by plating on minimal medium in the light. Between 10 and 27 colonies were obtained on each of the six transformation plates, compared to zero on the two negative control plates to which no plasmid had been added: the successful complementation and the phenotypes of the complemented cell lines (Figure4) indicated that A1-UV1 is indeed a *taa1* mutant. This was confirmed by partial DNA sequencing of *TAA1* in A1-UV1, which identified an amber (TAG) nonsense mutation at codon 633 of *TAA1*. This mutation may be visualized as a *Pvu*II restriction site polymorphism within the gene (Figure5).

As only one colony was obtained from the initial UV experiment, a larger-scale screen was performed using strain A1 to ensure that the result was replicable. Seventy-seven colonies were obtained from across four 5-FC selection plates, of which 22 colonies were picked at random and screened for light sensitivity; ten of these (45%) showed no or poor growth in the light and so were considered to potentially carry mutations in *TAA1* or other nuclear factors required for PsaA (and CrCD) expression. Four of the light-sensitive colonies (A1-UV11, A1-UV13, A1-UV16 and A1-UV22) were checked for CrCD and PsaA accumulation by Western blotting, and were found to lack both proteins (Figure4). Like A1-UV1, these four mutants were complemented by the *TAA1* plasmid (Table S1), and so are likely to be additional *taa1* mutants, although the molecular basis of these mutations has yet to be determined. The easy isolation of five new *taa1* mutants demonstrates the utility of *crCD* as a selection system, and encouraged us to investigate whether other ROGE mutants may be similarly isolated.

#### Isolation of tca1 and mca1 mutants by linking crCD to the petA promoter and 5′ UTR

Expression of the *C. reinhardtii* chloroplast gene *petA*, encoding the cytochrome *f* subunit of the cyt *b*_6_*f* complex, is known to require two *trans*-acting factors: MCA1 and TCA1. MCA1 stabilizes the *petA* transcript through the 5′ UTR (Loiselay *et al.*, 2008), whilst TCA1 is required for translation (Wostrikoff *et al.*, 2001; Raynaud *et al.*, 2007). MCA1 is also involved in the control by epistasy of synthesis (CES) process, whereby the synthesis of cytochrome *f* is down-regulated in the presence of unassembled cytochrome *f* subunits (Choquet *et al.*, 2003; Boulouis *et al.*, 2011). To test whether *mca1* and *tca1* mutants may be recovered using the *crCD* marker, a new transgenic *C. reinhardtii* line was prepared using a construct in which *crCD* had been fused to a copy of the *petA* promoter and 5′ UTR by one-step isothermal assembly (Gibson *et al.*, 2009). This line was named T1, and introduction of the marker into the chloroplast genome again conferred sensitivity to 5-FC.

**Figure 6:**
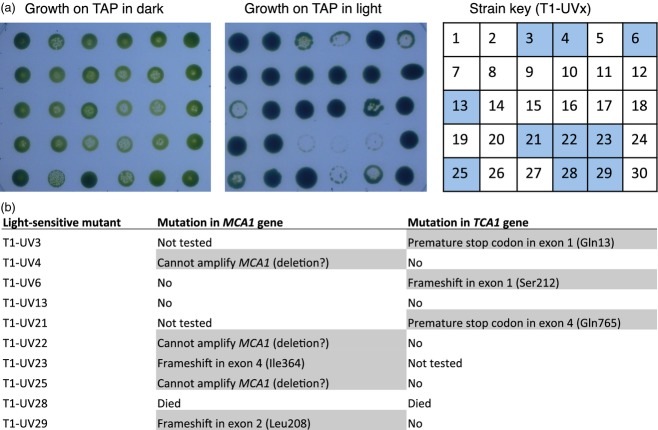
Isolation of *mca1* and *tca1* mutants using cell line T1, in which CrCD and cytochrome *f* are expressed under the control of the *petA* promoter and 5′ UTR. (a) Identification of light-sensitive UV mutants of T1. Thirty 5-FC-resistant colonies from the UV mutagenesis screen were spotted onto two TAP agar plates and incubated in the dark or under 50 μE m^−2^ sec^−1^ light. Ten of the mutants, indicated by shaded squares on the strain key (right), showed poor growth in the light, and were analysed by PCR and DNA sequencing of the *MCA1* and *TCA1* genes to identify any mutations. (b) Nuclear mutations identified in the ten light-sensitive UV mutants of cell line T1.

**Figure 7:**
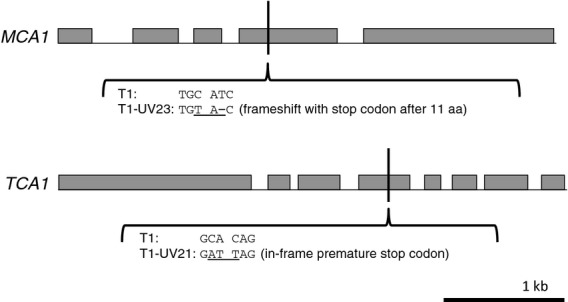
Examples of mutations recovered in the *MCA1* and *TCA1* genes. Exons are represented by grey boxes. The scale is shown at the bottom right; both genes are 4.1 kb.

As before, the T1 cell line was mutagenized using UV irradiation and plated in the dark on medium containing acetate and 5-FC to recover 5-FC-resistant mutants. A total of 1200 colonies was obtained across two selection plates. Of 30 colonies picked at random, ten (33%) were light-sensitive (Figure6a). Upon PCR amplification and complete DNA sequencing of the *MCA1* and *TCA1* genes (each 4.1 kb), five of these ten mutants were found to have premature stop codons or frameshifts in either *MCA1* or *TCA1* (Figures6b and 7). For three other mutants, *TCA1* was found to be intact but no sections of the *MCA1* gene could be amplified with any combination of the six primers normally used for analysis of *MCA1*. These mutants are therefore presumed to have large deletions or rearrangements at the *MCA1* locus.

**Figure 8:**
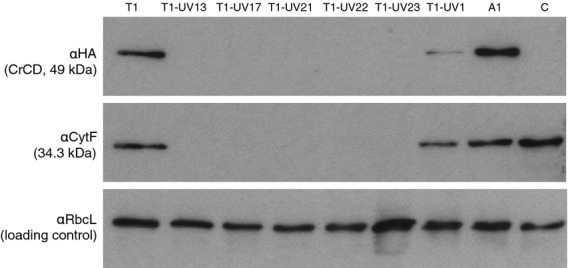
Western analysis of CrCD and cytochrome *f* protein expression in *C. reinhardtii* T1 and its UV mutants. Six UV mutants of T1 with various phenotypes and genotypes (see Figure6) were grown in liquid TAP medium in the dark, together with three control strains. Cell lysates were separated by SDS–PAGE on three identical gels, blotted and incubated with antibodies as indicated on the left. Strain C is an ‘empty vector’ control. Strains A1 and T1 contain the *crCD* gene under the control of the *psaA* exon 1 and *petA* promoter/5′ UTR, respectively.

The presence of CrCD and cytochrome *f* proteins was analysed in six of the UV mutants of cell line T1 (Figure8), representing the various phenotypes and genotypes that had been observed by growth tests and DNA sequencing. As expected, cytochrome *f* does not accumulate in mutants T1-UV21, T1-UV22 or T1-UV23 due to the lack of MCA1 or TCA1 to stabilize the *petA* transcript or initiate translation. Also shown in Figure8 are three 5-FC-resistant mutants with low, medium and high light tolerance (T1-UV13, T1-UV17 and T1-UV1, respectively) that do not have any mutations in *MCA1*, *TCA1* or *crCD*. Of these, T1-UV13 and T1-UV17 do not accumulate cytochrome *f* or CrCD. It is possible that these represent novel mutants affected in the expression of the *petB* or *petD* chloroplast genes that encode CES assembly partners of cytochrome *f*, as discussed below.

## Discussion

Expression of the modified and codon-optimized cytosine deaminase gene (*crCD*) in the *C. reinhardtii* chloroplast results in strains with an easily scorable or selectable phenotype of 5-FC sensitivity. As such, the marker represents a simple genetic tool for isolating resistant mutants that are defective in *crCD* expression, either because of mutations directly in the chloroplast *cis* elements used to drive expression, or (as shown here) in nuclear-encoded *trans*-acting factors that are specifically required for the functioning of these *cis* elements. The first class of mutants would provide important insights into key nucleotide sequences of chloroplast promoters, introns, 5′ UTRs and 3′ UTRs that are essential for function, and are distinguishable from the second class of mutant in which expression of both *crCD* and the endogenous chloroplast gene containing the *cis* element is affected.

As a proof of concept for recovering mutants of specific *trans*-acting factors, we focused on those known to bind two 5′ UTRs: those of *psaA* exon 1 and *petA*. In *C. reinhardtii*, the PsaA subunit of PSI is encoded by three separate exons that are independently transcribed, and the transcripts are spliced *in trans* to form the mature mRNA (Choquet *et al.*, 1988; Jacobs *et al.*, 2013). As many as 14 nuclear loci appear to be involved in the splicing of one or both of the split introns (Goldschmidt-Clermont *et al.*, 1990), with six factors identified to date (Jacobs *et al.*, 2013). In principle, it would be possible to screen for *trans*-splicing mutants using a split *crCD* marker. However, we sought new mutants of the one factor (TAA1) that is known to be required once the mature mRNA is formed, and which acts via the *psaA* 5′ UTR to mediate translation (M. Goldschmidt-Clermont, Department of Botany and Plant Biology, University of Geneva, Switzerland; personal communication). Our screen produced five mutants, all of which were complemented using a plasmid carrying the wild-type *TAA1*, suggesting that TAA1 is the only essential nuclear-encoded factor associated with the *psaA* 5′ UTR. The *taa1* Q633STOP nonsense mutation in line A1-UV1 leads to a lack of detectable PsaA protein accumulation, indicating that the truncated TAA1 protein is non-functional or unstable. The specific mutations in the other four cell lines were not determined; however, mis-sense mutants resulting in amino acid substitutions would provide new insights into key residues involved in the specific interaction of TAA1 with its cognate RNA. Immunoblotting with an anti-TAA1 antibody would help to identify such mis-sense mutants that accumulate a full-length (202 kDa) but non-functional factor.

The second screen targeted factors required for expression of the *petA* gene, encoding cytochrome *f*. It is believed that only two factors (MCA1 and TCA1) are involved in *petA* expression, with both factors acting on the 5′ UTR of the *petA* transcript and having partially overlapping but distinct functions (Boulouis *et al.*, 2011). MCA1 binds to the first 21 bases to stabilize the mRNA, while TCA1 binds further downstream and mediates translation initiation (Boulouis *et al.*, 2011). Our screen recovered two new *mca1* mutants, T1-UV29 and T1-UV23, with frameshift mutations in exons 2 and 4, respectively, together with three putative *mca1* deletion mutants. The mutation in T1-UV29 falls in the N-terminal region of MCA1, between the proposed transit peptide and the start of the PPR motifs (Loiselay *et al.*, 2008), while the frameshift in T1-UV23 occurs within the fourth PPR motif of MCA1, indicating that a truncated protein containing only the first three of 14 PPR motifs is unable to stabilize *petA* transcripts. Indeed, the missing PPR sequences are likely to be required to confer specificity of binding of MCA1 to the *petA* 5′ UTR using the ‘PPR code’ (Barkan *et al.*, 2012), and the C-terminal end of MCA1 is known to interact with TCA1 (Boulouis *et al.*, 2011). For TCA1, the structural domains are less well characterized, but analysis of the three *tca1* mutants T1-UV3, T1-UV6 and T1-UV21 in which nonsense or frameshift mutations occur at codons 13, 212 and 765, respectively, indicated that a truncated version with only the first 764 of 1103 amino acids is either unstable or non-functional. The *petA* screen also recovered two mutants (T1-UV13 and T1-UV17) that did not accumulate cytochrome *f* but were unaffected in either *MCA1* or *TCA1*. It is possible that these represent mutants affected in expression of the *petB* or *petD* chloroplast genes that encode the cytochrome *b*_6_ and subunit IV partners of cytochrome *f* in the cyt *b*_6_*f* complex. These subunits are involved in an intricate CES feedback mechanism for controlling cytochrome *f* levels, in which newly synthesized protein that does not assemble with its partners instead binds MCA1, leading to proteolytic degradation of MCA1 and hence destabilization of the *petA* mRNA (Boulouis *et al.*, 2011). This would explain the lack of both cytochrome *f* and CrCD in such mutants, as expression of *crCD* was under the control of the *petA* 5′ UTR.

Although the two mutant screens initially involved selection for 5-FC resistance, we also took advantage of the expected light-sensitive phenotype of PSI and cyt *b*_6_*f* mutants to identify the desired mutant classes. For the first screen, 45% of the mutants had a light-sensitive phenotype, while in the second screen, 33% of the mutants had a light-sensitive phenotype. It is likely that the 5-FC-resistant but light-tolerant lines are mutants that are affected either in the *crCD* expression cassette itself, or in nuclear genes encoding chaperones required for CrCD stability or enzymes that operate downstream of CrCD to convert 5-FU to more toxic compounds. We have yet to analyse these mutants. Nevertheless, the high frequencies with which *bona fide* mutants for the *trans*-acting factors were recovered suggests that a simple screen for 5-FC resistance is sufficient to recover such mutants even for chloroplast gene products where loss of the protein would not give an easily scorable phenotype such as light sensitivity, loss of photoautotrophy or major changes in chloroplast fluorescence kinetics. Such chloroplast gene products may include some of the minor subunits of PSII such as that encoded by *psbZ* (Swiatek *et al.*, 2001), or those encoding open reading frames of unknown function such as *orf58* (Takahashi *et al.*, 1996). In addition, the *crCD* marker may be combined with novel screens, such as that for a ‘yellow in the dark’ phenotype, in order to study the expression of the three chloroplast genes (*chlB*, *chlL* and *chlN*) involved in light-independent chlorophyll biosynthesis (Cahoon and Timko, 2000). Finally, the marker may be used to select for mutations in ROGE genes required for transcription of individual chloroplast genes or operons, or those required for post-transcriptional control of two or more separately transcribed genes (provided that the loss of expression of any of the chloroplast genes is not lethal). To date, such ROGE factors have not been described for the *Chlamydomonas* chloroplast.

In order to identify nuclear genes for previously unknown ROGEs, insertional mutagenesis may be used in place of UV or chemical mutagenesis prior to selection on 5-FC. In this technique, random integration of marker DNA into the nuclear genome results in a transformant population containing null mutants in which the marker has disrupted, and physically tagged, an unknown gene (Gumpel and Purton, 1994). Efficient nuclear transformation methods such as vortexing with glass beads or electroporation are well established for *Chlamydomonas*, and allow rapid generation of sufficiently large populations of transformants for selection of specific mutant phenotypes (Dent *et al.*, 2005). Methods to determine insertion sites include adaptor-mediated PCR and TAIL-PCR (Colombo *et al.*, 2002; Barbieri *et al.*, 2011; Ma *et al.*, 2011). After identification of the insertion site, the null mutant may be used to test the functionality of variants of the ROGE by complementation. Examples of ROGEs that are known to be required for expression of chloroplast genes, but for which the nuclear gene encoding the ROGE has yet to be identified, include MCB1 and MCG1; these stabilize the *petB* and *petG* transcripts, respectively (Wollman, 1998).

The optimized version of *codA* that we used worked well for isolation of the desired nuclear mutants using the *C. reinhardtii* cell line, expression cassettes and 5-FC concentration that we tested. However, to study weak chloroplast promoters/UTRs or to decrease the 5-FC concentration required for selection, it may be possible to adopt one of the enhancements to the *codA* selection system that have been developed for other organisms. In developing a markerless gene deletion system for acetic acid bacteria, Kostner *et al*. (2013) found that introducing the *E. coli* cytosine permease gene *codB* alongside *codA* decreased the minimum inhibitory concentration of 5-FC eightfold due to increased uptake of 5-FC. Another approach involves co-expression of a uracil phosphoribosyltransferase enzyme with *codA* to increase conversion of 5-FU to 5-fluorouridine 5′-monophosphate, enhancing the cytotoxicity of 5-FC (Tiraby *et al.*, 1998). A fusion protein of *Saccharomyces cerevisiae* cytosine deaminase, *Haemophilus influenzae* uracil phosphoribosyltransferase and the red fluorescent protein mDsRed was able to enhance the sensitivity of mammalian cell lines to 5-FC compared to those expressing only cytosine deaminase, and enabled imaging and flow cytometry (Xing *et al.*, 2008).

The optimized *crCD* gene provides a targeted tool for isolation of specific classes of nuclear mutants in *C. reinhardtii*. We suggest that the *codA* selectable marker systems used in other organisms may be improved by adopting the variant enzyme developed by Fuchita *et al*. (2009) used here, as it has a high affinity for 5-FC. The availability of a negative selectable marker for *C. reinhardtii*, and potentially other microalgae for which chloroplast transformation has now been demonstrated (Purton *et al.*, 2013), provides an opportunity to increase our understanding of the precise coordination between nuclear and chloroplast-encoded gene expression.

## Experimental Procedures

### *C. reinhardtii* strains and growth media

The recipient line used to create transformant lines A1 and T1 and control line C was TN72, a *psbH* deletion mutant of the cell wall-deficient strain *C. reinhardtii cw15*.3A (mt+) (T. Wannathong, R.Y., J. Waterhouse, C. Economou and S.P, all Algal Research Group, University College London, unpublished results). The cell lines are available upon request. *C. reinhardtii* cell lines were cultured on Tris/acetate/phosphate (TAP) plates containing 2% agar (Harris *et al.*, 2009) at 25°C. For liquid cultures, TAP medium in an Erlenmeyer flask of at least twice the culture volume was inoculated with a loopful of freshly streaked *C. reinhardtii* cells and incubated at 25°C, with shaking at 120 rpm. Both plate and liquid cultures were usually grown at light intensities appropriate to the strain: in the dark (foil-wrapped) for potential PSI or cyt *b*_6_*f* mutants, under low light (in a growth chamber at 50 μE m^−2^ sec^−1^ but wrapped in white tissue) for the PSII-deficient line TN72, and at 50 μE m^−2^ sec^−1^ for other cell lines. However, for strain comparison experiments (growth tests and Western blotting), all strains were grown under the same conditions, as described in the figure legends.

### 5-FC, 5-FU and light sensitivity tests

5-FC (product F7129) and 5-FU (product F6627) were purchased from Sigma-Aldrich (http://www.sigmaaldrich.com) and prepared as 10 mg ml^−1^ solutions in TAP medium. Both compounds are soluble in water, but TAP medium was used to prevent dilution of the TAP ingredients when preparing plates. For Figures1, 3 and 4, liquid *C. reinhardtii* cultures were adjusted to equal optical densities at 750 nm, and 5 μl of cells were spotted onto TAP agar containing 5-FC, 5-FU or no drug as appropriate. The position of each spot is indicated by a dot marked with a pen. For Figure6, a pipette tip was dipped in *C. reinhardtii* growth from an agar plate, and the cells were resuspended in 50 μl TAP medium. Then 5 μl of cells were spotted onto TAP agar as before. Plates were incubated at 25°C.

### Design of *crCD* and plasmid construction

An optimized version of the *E. coli codA* gene was synthesized by GeneArt (http://www.lifetechnologies.com) and named *crCD*. This incorporated three amino acid changes (V152A, F316C and D317G) that were shown by Fuchita *et al*. (2009) to increase the affinity of the enzyme for 5-FC, and was codon-optimized for the *C. reinhardtii* chloroplast using the program CUO developed by our laboratory (http://www.ucl.ac.uk/algae/Genetic_engineering_tools). The sequence of *crCD* is available in Appendix S1 and from the EMBL database under accession number LM643813. The accession number of the original *E. coli* gene product is NP_414871, and that of Fuchita *et al*.'s CodA protein is PDB 3G77_A. *CrCD* was inserted into the chloroplast expression vector pSRSapI using one-step isothermal assembly (Gibson *et al.*, 2009) and transformation of chemically competent *E. coli* DH5α using ampicillin selection (Sambrook and Russell, 2001) to create plasmids pRY127d and pRY128d, which have an HA tag and no tag on *crCD*, respectively. Insertion into pSRSapI (T. Wannathong, R.Y., J. Waterhouse, C. Economou and S.P, all Algal Research Group, University College London, unpublished results) places the gene of interest between a *psaA* exon 1 promoter/5′ UTR and an *rbcL* 3′ UTR, both from *C. reinhardtii*, with an intact copy of *psbH* in the downstream flanking sequence. One-step isothermal assembly was subsequently used to exchange the *psaA* exon 1 promoter/5′ UTR for that of *petA* to create plasmid pRY143a. The DNA sequences of the genetic elements are given in Appendix S1; the plasmids are available upon request.

DNA was amplified using Phusion high-fidelity DNA polymerase (Thermo Scientific, http://www.thermoscientific.com) according to the manufacturer's instructions. Plasmid DNA was extracted from *E. coli* cultures by alkaline lysis (Sambrook and Russell, 2001) for small preparations or using a QIAfilter Plasmid Midi kit (Qiagen, http://www.qiagen.com) for use in algal transformation.

### *C. reinhardtii* transformation

*CrCD* was introduced into the *psbH–trnE2* intergenic region of the chloroplast genome in *C. reinhardtii* line TN72 by homologous recombination of flanking DNA, restoring a functional *psbH* gene and the ability to grow photosynthetically. The transformation procedure was based on that described by Kindle *et al*. (1991). Cells (500 ml) were grown in TAP medium to a density of 2 × 10^6^ cells ml^−1^, harvested by centrifugation (3800 ***g***, 5 min, 16°C), and resuspended in TAP medium to 2 × 10^8 ^cells ml^−1^. Then 300 μl of cells, 10 μg of circular plasmid DNA and 0.3 g of glass beads (Sigma-Aldrich, diameter 0.4–0.6 mm) were vortexed in a glass test tube for 15 sec; 3.5 ml high-salt minimal medium (Harris *et al.*, 2009) containing 0.5% agar was added, and the mixture was poured onto an high-salt minimal medium plate containing 2% agar. Plates were incubated at 25°C under 50 μE m^−2^ sec^−1^ light for 2–3 weeks. Colonies were checked for homoplasmicity of the transgene by PCR. Nuclear transformation followed the same protocol but using 0.5 μg circular plasmid DNA per plate.

### UV mutagenesis

A 30 ml culture of *C. reinhardtii* cells was grown in TAP medium to an OD_750_ of 1.0 (48 h), and poured into a plastic Petri dish containing a sterile metal paper clip. A magnetic stirrer was used to prevent settling. Cells were subjected to UV irradiation (254 nm, 6 W) for a pre-determined time to achieve 10% survival (4 min), then incubated in the dark for 1 h to prevent photoreactivation and to allow some time for the CrCD protein to degrade before selection. Then 125 μl of cells were mixed with 7 ml of 0.5% TAP agar and 5-FC (2 mg ml^−1^ final concentration), and poured onto a 140 mm diameter/62 ml TAP agar plate containing 2 mg ml^−1^ 5-FC. Several plates prepared in this way were incubated in the dark at 25°C for 3 weeks. Colonies were streaked onto TAP agar and incubated in the dark.

As we wished to isolate mutant cells that do not convert 5-FC to toxic 5-FU from a pool of cells that do initially produce (and release) 5-FU, it was important to minimize the bystander killing effect by not plating the cells too densely. We found that using the plating density above allowed the desired mutants to survive.

### Western blot analysis

For Western blot analysis, 10 ml mid-log phase *C. reinhardtii* cultures grown in the dark were harvested by centrifugation (4000 ***g***, 5 min) and resuspended in 0.8 m Tris/HCl pH 8.3, 0.2 m sorbitol and 1% β-mercaptoethanol to equal cell densities, as measured by light scattering of the original culture at 750 nm. Then 15% acrylamide SDS–PAGE gels were prepared in a Mini-PROTEAN Tetra cell system (Bio-Rad, http://www.bio-rad.com) according to the manufacturer's instructions, and run at 150 V for 90 min. Proteins were transferred to a Hybond ECL nitrocellulose membrane (GE Healthcare, http://www3.gehealthcare.co.uk) using a Trans-blot semi-dry transfer cell (Bio-Rad) run at 20 V for 1 h. After overnight blocking in TBS with 0.1% Tween (TBS-T) and 5% milk, membranes were incubated with the primary antibody for 1 h, washed in TBS-T for 30 min, incubated with the secondary antibody for 1 h, and washed again in TBS-T for 30 min, all at room temperature. SuperSignal West Pico Chemiluminescent substrate (Thermo Scientific) and Hyperfilm ECL (GE Healthcare) were used for detection. The primary antibodies used, all produced in rabbit, were αHA (Sigma-Aldrich product H6908, 1:5000 in TBS-T), αRbcL (a gift from John Gray, Department of Plant Sciences, University of Cambridge, UK; 1:20 000 in TBS-T + 0.5% milk), αCyt*f* (a gift from Francis-André Wollman, Institut de Biologie Physico-Chimique, Paris, France; 1:20 000 in TBS-T) and αPsaA (a gift from Michel Goldschmidt-Clermont, Department of Botany and Plant Biology, University of Geneva, Switzerland; 1:200 000 in TBS-T + 0.5% milk). The secondary antibody for all blots was ECL α-rabbit IgG horseradish peroxidase-linked antibody from donkey (GE Healthcare product NA934, 1:5000 in the same buffer as for the primary antibody used).

### *C. reinhardtii* DNA extraction

Total genomic DNA was extracted from *C. reinhardtii* using a method based on that described by Rochaix *et al*. (1988). First, 10 ml of a mid-log culture in TAP medium was harvested by centrifugation (4000 ***g***, 5 min) and resuspended in 0.35 ml TEN buffer, pH 8.0 (50 mm EDTA, 20 mm Tris/HCl, 0.1 m NaCl). Then 50 μl pronase (8 mg ml^−1^; Roche, http://www.roche-applied-science.com) and 50 μl of 10% SDS were added, and the sample was incubated at 55°C for 2 h. Next, 2 μl diethyl pyrocarbonate was added, and the sample was heated at 70°C for 15 min. After addition of 50 μl of 5 m potassium acetate, the lysate was left on ice for 30 min and cell debris was pelleted (21 000 ***g***, 15 min). The supernatant was mixed with an equal volume of phenol, and centrifuged (21 000 ***g***, 2 min). The upper aqueous phase was isolated, and 800 μl of 100% ethanol was added to precipitate the DNA, which was then pelleted by centrifugation (21 000 ***g***, 20 min). The DNA pellet was washed in 70% ethanol, air-dried and resuspended in 50 μl distilled water.

### DNA sequencing

To confirm plasmid constructs and to identify mutations in particular nuclear genes in the *C. reinhardtii* UV mutants, Sanger sequencing was performed on plasmids and PCR products by Source Bioscience (http://www.sourcebioscience.com). The primers used to amplify and sequence *TAA1*, *TCA1* and *MCA1* are given in Table S2. When a mutation was identified, the gene fragment was PCR-amplified again and re-sequenced to ensure that the mutation was genuine.

## Supporting Information

Figure S1 Analysis of transgenic cytosine deaminase expression in the *C. reinhardtii* chloroplast using non-optimized genes.

Figure S2 PCR analysis of *C. reinhardtii* chloroplast transformants, demonstrating correct integration of foreign DNA and homoplasmy.

Table S1 UV mutants of *C. reinhardtii* cell line A1 may be complemented with the *TAA1* plasmid.

Table S2 Primers used for identification of mutations in nuclear genes.

Appendix S1 DNA sequences used in this work, and the translated sequence of the synthetic *crCD* gene.
