# Supplementary material for: Cytosine deaminase as a negative selectable marker for the microalgal chloroplast: a strategy for the isolation of nuclear mutations that affect chloroplast gene expression
Source: Plant J. 2014 Sep 18;80(5):915–25. doi: 10.1111/tpj.12675 (PMC4282525; doi:10.1111/tpj.12675)
Supplement: Figure S1 — Analysis of transgenic cytosine deaminase expression in the C. reinhardtii chloroplast using non-optimized genes. [file tpj0080-0915-SD1.docx]

**(a)**


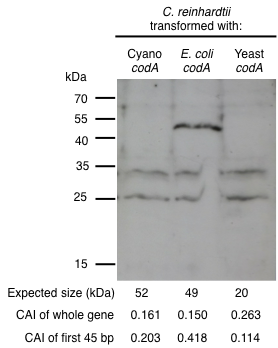


**(b)**


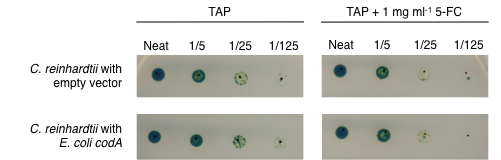


**Figure S1. Analysis of transgenic cytosine deaminase expression in the *Chlamydomonas reinhardtii* chloroplast using non-optimized genes.**

*C. reinhardtii* strain TN72 was transformed with cytosine deaminase genes amplified from three different organisms. The genes were under the control of the *C. reinhardtii atpA* promoter and 5’ UTR and *rbcL* 3’ UTR, and were targeted by homologous recombination into the *psbH* region of the chloroplast. All had a haemagglutinin (HA) tag sequence at the C-terminus. The genes were from the cyanobacterium *Synechocystis* sp. PCC6803 (labelled as ‘Cyano *codA*’; protein accession no. BAA17527), *Escherichia coli* (accession NP_414871) and *Saccharomyces cerevisiae* (labelled as ‘Yeast *codA*’; accession NP_015387).

**(a)** Western analysis. The blot was probed with an αHA primary antibody and ECL secondary antibody for chemiluminescent detection. Only the *E. coli* CodA enzyme (49 kDa) could be detected. Non-specific bands are present at approximately 25 and 33 kDa in all lanes. CodA protein expression did not correlate with the codon adaptation index (CAI) of the transgenes, calculated with respect to highly-expressed genes in the *C. reinhardtii* chloroplast, but *E. coli codA* does have a higher CAI across the first 45 bp than *codA* from the other two sources. Codon usage near the 5’ end of a gene is thought to be particularly important for translation (Goldman *et al.* 1995).

**(b)** Demonstration that the natural *E. coli codA* gene (under the *atpA* promoter) does not confer sensitivity to 5-FC upon *C. reinhardtii*.

**Goldman, E., Rosenberg, A.H., Zubay, G., Studier, F.W.** (1995) Consecutive low-usage leucine codons block translation only when near the 5' end of a message in *Escherichia coli.* *J Mol Biol*, **5**, 467-473.
