# Supplementary material for: Cytosine deaminase as a negative selectable marker for the microalgal chloroplast: a strategy for the isolation of nuclear mutations that affect chloroplast gene expression
Source: Plant J. 2014 Sep 18;80(5):915–25. doi: 10.1111/tpj.12675 (PMC4282525; doi:10.1111/tpj.12675)
Supplement: Figure S2 — PCR analysis of C. reinhardtii chloroplast transformants, demonstrating correct integration of foreign DNA and homoplasmy. [file tpj0080-0915-SD2.docx]

**(a)**


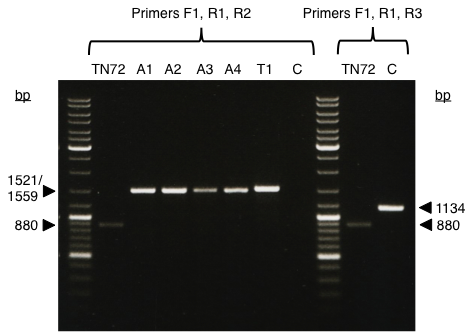


**(b)**


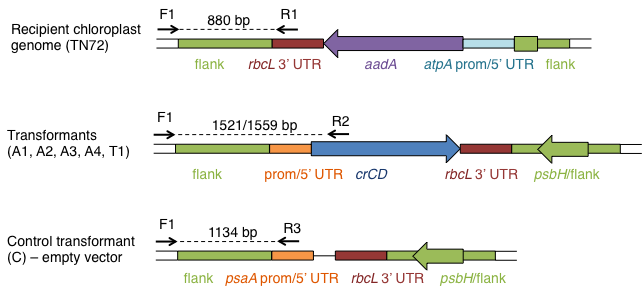


**Figure S2. PCR analysis of *C. reinhardtii* chloroplast transformants, demonstrating correct integration of foreign DNA and homoplasmy.**

**(a)** Agarose gel electrophoresis of PCR products. Template DNA was used from the strains listed above each lane. Three primers were added to each reaction; chloroplast genomes with a parental (TN72) gene layout would lead to an 880 bp product from primers F1 and R1, whereas transformed chloroplast genomes would lead to a 1521/1559 bp product from primers F1 and R2 (left) depending on the size of the promoter/5’ UTR element, or a 1134 bp product from primers F1 and R3 (right). The absence of an 880 bp product in the transformant lanes indicates homoplasmy. Unlabelled lanes contain GeneRuler DNA Ladder Mix (Thermo Scientific).

**(b)** Representation of primer binding positions on the recipient and transformed chloroplast genomes.

Primer sequences were as follows (5’-3’):

F1 GTCATTGCGAAAATACTGGTGC

R1 CGGATGTAACTCAATCGGTAG

R2 GTAGCATCAGAAACGTCTACGTG

R3 CATggatttctccttataataac
