# Supplementary material for: Cytosine deaminase as a negative selectable marker for the microalgal chloroplast: a strategy for the isolation of nuclear mutations that affect chloroplast gene expression
Source: Plant J. 2014 Sep 18;80(5):915–25. doi: 10.1111/tpj.12675 (PMC4282525; doi:10.1111/tpj.12675)
Supplement: Table S1 — UV mutants of C. reinhardtii cell line A1 may be complemented with the TAA1 plasmid. [file tpj0080-0915-SD3.docx]

**Table S1. UV mutants of *C. reinhardtii* cell line A1 can be complemented with the *TAA1* plasmid.**

Selection was carried out on HSM (minimal medium) in the light; integration of the *TAA1* plasmid into the nuclear genome restores phototrophic growth in all four of the cell lines tested.

| ***C. reinhardtii* cell line** | **No. of colonies on transformation plates (*TAA1* plasmid) (n=2)** | **No. of colonies on no-DNA control plates (n=2)** |
| --- | --- | --- |
| A1-UV11 | 39, 47 | 0, 1 |
| A1-UV13 | 15, 25 | 0, 1 |
| A1-UV16 | 24, 33 | 1, 2 |
| A1-UV22 | 28, 28 | 0, 0 |
