# Supplementary material for: Cytosine deaminase as a negative selectable marker for the microalgal chloroplast: a strategy for the isolation of nuclear mutations that affect chloroplast gene expression
Source: Plant J. 2014 Sep 18;80(5):915–25. doi: 10.1111/tpj.12675 (PMC4282525; doi:10.1111/tpj.12675)
Supplement: Table S2 — Primers used for identification of mutations in nuclear genes. [file tpj0080-0915-SD4.docx]

**Table S2. Primers used for the identification of mutations in nuclear genes.**

Genes were amplified from *C. reinhardtii* genomic DNA in sections using Phusion Polymerase, GC buffer and 3% DMSO (all Thermo Scientific). 320 ng template DNA was used per 25 μl reaction. Products were cleaned using a PCR Purification Kit (Thermo Scientific) before DNA sequencing.

| **Purpose** | **Primer sequences 5’ to 3’** |
| --- | --- |
| Amplify and sequence/digest 820 bp region of *TAA1* | ACGGCGGCTGCTGAGCTGT |
|  | CGGAGAACAGAACGGAGGGC |
| Amplify and sequence whole *TCA1* gene | GCACGATCTAATCAGTGTAGC |
|  | AGTTTGGGAGACACGCTTG |
|  | GACAATGACGTTAATGCGTACC |
|  | CATGTCGTACAGGGCATATG |
|  | CACCAGCCTCAACAGCACG |
|  | GTGGCAGCCGCACCAGTGG |
| Amplify and sequence whole *MCA1* gene | CTGCATCCTTACTTGCAACG |
|  | AACAGATCGTCGGAAACACG |
|  | TGGACAAGCTGGTGCTGAG |
|  | AGCACCTGGCGCTCGATAG |
|  | GGTCCCTTGACCGGTTACTG |
|  | TGGACGCCGCAGTCCATAT |
