# Supplementary material for: Cytosine deaminase as a negative selectable marker for the microalgal chloroplast: a strategy for the isolation of nuclear mutations that affect chloroplast gene expression
Source: Plant J. 2014 Sep 18;80(5):915–25. doi: 10.1111/tpj.12675 (PMC4282525; doi:10.1111/tpj.12675)
Supplement: Appendix S1 — DNA sequences used in this work, and the translated sequence of the synthetic crCD gene. [file tpj0080-0915-SD5.docx]

**APPENDIX S1. DNA and translated sequences used in this work.**

**DNA sequence of the synthetic *crCD* gene.**

Amino acid changes (compared to the natural *E. coli* CodA enzyme) V152A, F316C and D317G are shown in turquoise. HA tag is shown in red.

ATGTCTAACAACGCTTTACAAACAATTATTAACGCTCGTTTACCAGGTGAAGAAGGTTTATGGCAAATTCACTTACAAGACGGTAAAATTTCAGCTATTGATGCTCAATCTGGTGTAATGCCAATTACTGAAAACTCTTTAGATGCTGAACAAGGTTTAGTTATTCCACCATTCGTTGAACCACACATTCACTTAGATACTACACAAACAGCTGGTCAACCAAACTGGAACCAATCAGGTACTTTATTTGAAGGTATTGAGCGTTGGGCTGAACGTAAAGCTTTATTAACACACGACGACGTTAAACAACGTGCTTGGCAAACATTAAAATGGCAAATTGCTAACGGTATTCAACACGTACGTACTCACGTAGACGTTTCTGATGCTACTTTAACAGCTTTAAAAGCTATGTTAGAAGTTAAACAAGAAGTAGCTCCATGGATTGACTTACAAATTGCTGCTTTCCCACAAGAAGGTATTTTATCATACCCAAACGGTGAAGCTTTATTAGAAGAAGCTTTACGTTTAGGTGCTGATGTTGTTGGTGCTATTCCACACTTCGAATTTACACGTGAATATGGTGTTGAATCTTTACACAAAACATTTGCTTTAGCTCAAAAATATGATCGTTTAATTGATGTTCACTGTGACGAAATTGATGACGAACAATCACGTTTCGTTGAAACAGTAGCTGCTTTAGCTCACCACGAAGGTATGGGTGCTCGTGTTACTGCTTCACACACTACAGCTATGCACTCTTACAACGGTGCTTACACTTCTCGTTTATTCCGTTTATTAAAAATGTCTGGTATTAACTTCGTTGCTAACCCATTAGTAAACATTCACTTACAAGGTCGTTTCGATACTTACCCAAAACGTCGTGGTATTACACGTGTTAAAGAAATGTTAGAATCAGGTATTAATGTTTGTTTTGGTCACGACGACGTTTGTGGTCCTTGGTACCCTTTAGGTACTGCTAACATGTTACAAGTTTTACACATGGGTTTACACGTATGTCAATTAATGGGTTACGGTCAAATTAACGACGGTTTAAACTTAATTACTCACCACTCTGCTCGTACTTTAAACTTACAAGACTACGGTATTGCTGCTGGTAACTCAGCTAACTTAATTATTTTACCAGCTGAAAACGGTTTCGATGCTTTACGTCGTCAAGTTCCAGTACGTTACTCAGTTCGTGGTGGTAAAGTTATTGCTTCAACTCAACCAGCTCAAACAACTGTTTATTTAGAACAACCAGAAGCTATTGACTACAAACGTTACCCATACGATGTTCCAGATTACGCTTAATAA

**Translated sequence of the synthetic *crCD* gene.**

Amino acid changes (compared to the natural *E. coli* CodA enzyme) V152A, F316C and D317G are shown in turquoise. These amino acids are numbered according to the substitutions in Fuchita *et al*. (2009) *Cancer Res*, 69, 4791-4799. HA tag is shown in red.

MSNNALQTIINARLPGEEGLWQIHLQDGKISAIDAQSGVMPITENSLDAEQGLVIPPFVEPHIHLDTTQTAGQPNWNQSGTLFEGIERWAERKALLTHDDVKQRAWQTLKWQIANGIQHVRTHVDVSDATLTALKAMLEVKQEVAPWIDLQIAAFPQEGILSYPNGEALLEEALRLGADVVGAIPHFEFTREYGVESLHKTFALAQKYDRLIDVHCDEIDDEQSRFVETVAALAHHEGMGARVTASHTTAMHSYNGAYTSRLFRLLKMSGINFVANPLVNIHLQGRFDTYPKRRGITRVKEMLESGINVCFGHDDVCGPWYPLGTANMLQVLHMGLHVCQLMGYGQINDGLNLITHHSARTLNLQDYGIAAGNSANLIILPAENGFDALRRQVPVRYSVRGGKVIASTQPAQTTVYLEQPEAIDYKRYPYDVPDYA

***C. reinhardtii psaA* exon 1 upstream element (promoter and 5’ UTR).**

AAGCTTTCTTAATTCAACATTTTTAAGTAAATACTGTTTAATGTTATACTTTTACGAATACACATATGGTAAAAAATAAAACAATATCTTTAAAATAAGTAAAAATAATTTGTAAACCAATAAAAAATATATTTATGGTATAATATAACATATGATGTAAAAAAAACTATTTGTCTAATTTAATAACCATGCATTTTTTATGAACACATAATAATTAAAAGCGTTGCTAATGGTGTAAATAATGTATTTATTAAATTAAATAATTGTTATTATAAGGAGAAATCC

***C. reinhardtii petA* upstream element (promoter and 5’ UTR).**

TATAAAGATCTTCCATGCATGAACTATGCTTTATTTGCTAAAAAAAAGATATAATATATGTTGAGAAGAAAAAAAATAAAATTTAAATAGTAATTATGAATGTAAATTACTTATGCTTACTTTTTCAGCTAGAATAAACTTGTTGAGGCTGCTTAGTTGCACTTTCCTCGTACCCGTATGGGTAGGGGTTTATCCACGAAAAAATTCTATTTTAAAATAGGAGTCCAGTTGAAAAGCAACTGGAATCCCCTTATAGATAAATTAATATCTATTTTAAAATTGAATAGTTTTTATTCTAGTTTCGTTTTAAGATTAATAAAATT

***C. reinhardtii rbcL* 3’ UTR used with *crCD*.**

tttttatttttcatgatgtttatgtgaatagcataaacatcgtttttatttttatggtgtttaggttaaatacctaaacatcattttacatttttaaaattaagttctaaagttatcttttgtttaaatttgcctgtctttataaattacgatgtgccagaaaaataaaatcttagctttttattatagaatttatctttatgtattatattttataagttataataaaagaaatagtaacatactaaagcggatgta

***C. reinhardtii TCA1* gene (start to stop codons)**

The *TCA1* gene in *C. reinhardtii* cell line T1 (this paper) is identical to that given on Phytozome v9.1 (www.phytozome.net) on chromosome 9, position 7684564 to 7680427:

ATGCTGCTGTCGCTGCAAAATGGAACAGCTGGCCTCCAGTTGGGATGTTCACGGCACCCGGGGGCGCTGCACCGCACTTGTGGCCGCGCGGCGACCGCATCCACGCCGTTGGACTGGCCGCTGAGCGATTCTGCGCAGCAGGATGCTCTGCAAGGTGCTGTCAATGAGGGACTGCGAGAGCGACCTGCACGAGCTGTATCAACCGGCCCCGACCAGGCTGCGGGAATTCGGCCACGCCTTATCCCCCCTCGTCGACGCCAGCCGCAACATCGCGCCCTCCGCGCGGACGAGGGCCGTACACAGCAGGCATCCAGCACTACCGACGGCCCATCCACCAGTGGCCGCGAGCACTACACTGCGGCTTCTGCGCCATGGCCGCAGCCGCAGGGAAGGGCACGCATCATGCCGCTACCCCGCTCCTCGCCTGCCGCCTCCACGTCCTCCATGGACGTGGAGCTGGAGGCGCTCATCTGGGCCATTGCTTCGGCACGTGGAAGCAGTGGTAACAGCTGGAGCACGAGTGGGCGCGGCAGCAGCGGCAGCACCAGCCTCAACAGCACGGGCGGGATTGGCCCCAGCGCGGCCGCTGCGGCGGCCGTCGGCGGTCCGGCACAGCACAGCTGGACGCCGGATTCGCTGCTGGCCGCACTTGACTTGGCGCTACCAGACATGGCACGGACGCGGCGGTCTGAAGTGGCGGCGCAGCAAGGAGACTATTCGAGCGCGGCCTGGAGCGACACCGGTGGCGCTGAGGATGCCAGGAGCAGGAGCCATAGCGGCAGCGGGCTGGCCTCGGTGCTGCTGCCGGCACTGGTGGCGCCCGCCTCCAACCTGCACCCGCCGTCATCCAGCTTAACGTGGTCTCCCGTGGGCGTGCCAGAAGAAGTGAGCAACTGGCTGGACGCGAGTGCAGTCCATGCAGACCTGGGCGAGGCGCGCAGCGACACTGCTGGCGGCAGCGGGCACGGCAGCGCCGTACTGGCGGGCCCGGGGTCAAGCCCTCACGGACCCCTGCCGCAGCCCACGTCTTTCCTTTCGTCGCAGACTCGGGGCTCACCGACGCCCTGGCGAGGAGCTGAGCCAGGCCTTGCTGACGCGGGTAGTGTGCAGCTGATGGGCCCACTGGCTGCTGAGGGGCCGGCGCTGGCCAGCCTGATGTGCGCGGCGGCCGGCGTGGCCTGGCTGACCCCGACCCAGCAGCAGCAGCAGGCTGCAGGCGACTGGGGACGAGCCCCTTCTGCAACAGTACTTACTCACACCGCAGTAACGTCGTCATTCAATGCGCTGGACCCGGCGTTGGAATGGGCGCTCGCCGTGCTCAGCACCCGGGTCTCGCCGGCTGAGCCCGCGGCGGCTGATGTGCGCCTGGCCAACCGCACCTTCGCCGCGCACGTCCGCCGCGCTGGTGGGCTTGACGCGGCGCGGCCAGAGTCGCATACACACGTGTTTTCGTCCGGCGTACCGGCTGCTAGGCAGGCTCCATCGACTGCTCTGCCATGGCAGGTGCTGCAGCCGCTGCTGACGGCGGTGGGCAACCGCCTGAGCCCGTGCTCGGCCCTGGCGCTGCTCCAGGTGCGAGCACTGGCGTGATGACCCCGGTTACCGAATGTAGTCAGTTTGGGTGTTGGCCTGGCTCGACCCACACAATGTTTTGTCGACTTGGTTGATTTGCTGGCATGGTGCGCATGGCCTAACTGCCGTGGGACCGTCATTGCCTCCTGCAGGTGTTGGTACGGCAGGTGGGCAGCAGCTGGCGACAGCAGCAGCCTGGGGACGCCTCCTCCCCTCTGTCCCACCGACAGCAGCAGCAGGTGGCGGCCACATGCCAGGCGCTATTTGCGGCGGCGCTCCGGCGGCCGGGCCAACTGCCGCGATCGGTGAGGATTGCGGACAATGACGTTAATGCGTACCTGGCACCGGTGAGGCGAGCAAACAACACCCACTATCATCTCCTGCTCGACTCGCAGACTGCCGCGGGGCTGCTGGTGGTGCTGCGGCGGCTGCAGCTGCACTCGCCGCCTACCTGCGCGCTGCTGGTAGAGCGGCTGCTGGCGGGACCTGGTGTTGCGGAGGTTGCAGGTGGCGTTGCGGCTGATCCTGGATCCACTGCTAAGTTGGAGGCAAACAGAAAAGGCGGCAAGCGGCTGCGGCTGGCGCTGCGCCACGGCCATGGCTGGGGGCGCGGCGTCACGCCTCTGCGGCTGGAGTGGGTGGCCATGGCCCTGTCCTGCCTGGCCCAGCTGCGGGTGCCGGCTGAGGCCCTGCCGCGGAGGACGGTGACGCGGCTGTTCAGGTGGGTGCACGACGCGCGCGGGTCTCTTCAAGCGTGTCTCCCAAACTGTACATGCCACACAGAGTGAAGCACGCCACTGGCGGGTAGCACGCATGCGGCGGGAAAGACAAAGACGACGCCACCGCTACGTTTGGCTGACCTGTGTCCTACTGCCCCGCTCGCAGGAGCGCTGTTGCGCCCGCCAACCGCCAGCGCTGGACGCCCTCGCTGGCCGCCGCCCTGCTGGTGTGGGCAGCTCGGCTGCACTGCCGCCCGCCCGCCGGCGCCCTGCAGCTGTACATGACCGGGCTGGTGGAGGTGCGGAGCGGGGCGTCGCGGCTGGTGCGGCTGCGGCGGCGTGCCGGCACGGGCACCGGCAGCAGCCCCACGGGCTCCATGGACAGCGCAGCCACCTCGTCACCGGCACAGCCCTGGAGCCTGTTGGCCCGCTTGCGCGGTCCCCAGTTGATGGCGGTGCTGGAGGCGCTGTGGCGGCTGTACGGGCCGGAGGCATCGACGCAGGCGGCAGCAGCTGCAGCGGCGGCAAGCGGCAGGAAGGCCTTGTCGCCTGTTTCGGGACCGCCACACAGCTTTCAGGTATGGTGCGCCGGTGTCGGCAAGAGCACACATCACAATATCTATGAATGCGACATTACAATAAGGGATATATATGCTGTTTCACCAGCTGAGCGTTGCCATGTCCTTGTGCGCCATCCATCTCTCGCAGCGTCGTCTCGCGGCCGCGCTGATGGGTCGGCTGCACCAGCTGCCCCGCCAGCAGCTGTTCCGACTGCCGCTGCTGCTGGCGCGGCTGGGGCTGACCAGCCTGCGGCAGCCGGACCACCTGCAGGTGAGTGCAGCATGGCGCTTACCTGACACGTCTCGCTGAGGTGCGTGCGATGCCGTTGTTTGGACAGTTTTCTCATCCGTCTATCGCTTCTGCCGCCGTACAGGCCTTCTCCGCACGGGTGCAGCAGGTGGCGGGCGGCTGCGACGCGCTGGACGCAGTGCTGCTGCTGCGCGGACTGGCGGCGGCAGCGCGGGACGAGTTGCAGACGTCTCACGGCGCGACACCTGCGTCCGGGTACTGCGGCTGGCAGCGCGGCGAGCTCGCGGACTTGGCCCTGCGGCGCTACACGGTGCGCTCAATGGAATCGTGGATTGTGAACACCTCCTTTTGGGTGCGCTTTGTTGTTTTAAGCTAGTCCGTCGCTGTCTGCAGGTGCTGCTACCCTCAGCGAGTCCGCGCTTCTCAGCCGCCGCCCTGCAAACACTGCCATTGCTGTGGCCGCCGCTGGACGTGGCTGCTGCGACCGCCGCTGCCCCCACTGGTGCGGCTGCCACCTCGGCCGAGGCTGACCTGCAGCGGCGGCGCGCCGCGGACACGGCGGCTGAGCGGCAGTGGCTGCTGCAGCAGCTGGACGACCACCTGGCGGAGGCGCTCGCGGCGGAGGCGGACCGGCTGGCGCAGGCGGCGGCGGAGGCGGCCAGCCGGCGCGGCAGCCGGCGCGGCAGTCGCAGAGGGCCCCGCAGGTTGGAGCACGACACGGGTGTGGGCCAGCTGGTGCTGCAGGTGCGTGGCGACAACCTGGCGCAGGGCTGGTGCATGCCGAGTTGCCGATCCCTCGCTGCAGAGGCACGTACCGGTAACACCTTATCGCATGCACACGCACGCAGGTCCTGGAGTCGCACGTGGCTCTGCGGCGGCGGCCCTCTCCACGGCTGCAGGCAGCGCTGGAAGCCGCGGTGGTGGCGGTGGCACCCGCACTGGGAACTAATGGCTGCGCACGTGTGCGGGCCGCGTACTCGGGTCTGGGTCTGAGTGCGGGTGGCCGCCTGAGTGTGGCCCTGGTCACGTTTGGGTGA

***C. reinhardtii MCA1* gene (start to stop codons)**

The *MCA1* gene in *C. reinhardtii* cell line T1 (this paper) is identical to that given on Phytozome v9.1 (www.phytozome.net) on chromosome 8, position 16828 to 20887:

ATGCCTATCACTGCAAGGCCGCCTATTTCCGCTGCGGCCGGTGCCAGCGGCACGGCTGGCAGCCTTCCTGCCCGTGTTACTGGTCCTGGTCGTCAGAACCAGGGCCACAATGCCCAGCACGTCCCCGTCGCGCGGGCTCCGACACTGGCGTCCGCGCGCTCTACCACGCGGACTTCGGCGGCGTCGGGTACCACCGTTATGACTGTGACGGACCGTGTTCGCAAGTCTATTGAGCGGCGGTCCATGGACGCTCGTCCGCAGGAGCAGGTGAGCTTTTTGTCGCGATGCCGCTCTTTTATGTGGCGATGCAACGAGCTTCGTAGGGTTGTCGTGCGTGAGCACGCTCGGTGCCCTATGATGTAGATTTAAGGTTGTTACCGCGGCAAGATCTAAGGTCCCTGGACCCGAACAGGTGTTGCAGCCGTTGTTGAACGTGCGTTCAACGCCACGCCACGCAACCGCCTCAACAGCGGTTGGGTTACCTTCAGCGCAGTTTGCCTGACTCGCCACGCAGCCACACGCCTGCCATTTTGCGTTTCGATGCACATGTATTGATAAGTCACCTAAATGCATTTTGCCGCTGCCGCCTGCCACTGTGTCCGCAACCGCAGGGTGAGCCTCTGGAGCCTCTGGAGCAGGTGCCCGAGGACCCGCTGGAGCGCCTCACCGCCGACCTGGCCGCGCTGTCCATGCAGCACGCCGCGGCTGTGGCCGCCGCTGTCGCTATCACCACCGCCTCCGCCTCAAGCTCGGGCACCTCCTCCTCCGCGCCTGCCCTGCCTGCGGCCTCCTCTGGCCGCAATGGCCGGCCTCAGGGCCCCAGCGGCCGCTACGGCTCCTCCTCCGCCTCGGGCC

CGGCGCCCGTGCACGCCAGCTACAACAACAGCGGCGCGCCGTCCTCATCTCCGAATGCGCTGCAGCAGCAGCAGTCTCAGTCTGGCTCCGCGGACAGTCGCCGCTGCGACCTGGGAGACACCGGTGAGTGCTGAGATTGGGGTGACGGTGTACTACTGCTGATCGGATCTGGGTATGTCAGCCCCCTCAAGTTGTTCTCGGTGATCTGTCAGCTTCCTAACCCAACCGTGTTCCCTTCTATCTCCGCCCTGCAGGTCTGCCCGCCCAGCTGCTGGCCCTGCGCGACCCCGCCTGCCCCGTGTCGCTGCCTCCTCCCACCGGCCTGCCCTCGTCCTGCCTGCCGCTGGACAACTGGAAGCTGGACAAGCTGGTGCTGAGCCTGTCCGCCAACAAGGCCACCTGGCGCCGCTCGCTGCTGTTGTTTGAGTGGCTCAAGGCCGCCGGGCACCAGCTGGATGACCGCCTGTGCACCACGGTGAGTGCGCACACAGGCCTTGGCGGTGGTGGCGGCCAGAAACATGTTGGACTGCCGACGGATGATTTTGTGGCGTGTTTCCGACGATCTGTTCCTTGCCTCGTGTTCTGACTTGCCTCGGTTCTCTGCCCTTTTCATTACGTGCCATGCAGCTGATCCGCGTGTGCAGCGACCACGGCGACGCTGTGTCGGCCCTGGCGGTTTACGACTGGATGACGGGCTCCACGGCCAGTGGCGGCGCGGCGCTGGAGGCCACTGCCTACACCTACACCGCGGCCATGCGCGCCGCGCTGGCGGGCGGCCTCACGGACCGTGCCCTCAGCATCTGGAACGAGGCCTGGCGCCGCCACAGCGCTGGGCGCCTGCAGCTGGACTGCCGGCTGTGCATCACCTACCTGGAGCTATGCACGCGGCTGGGCCTGACGGACCAGGCTCTGGCCATGTACGCGGCCATGCGCGCCGCGCCCGCCGGCAGCCGCATGGCGCCCACCGTGCATGCCTACACCGCCGCCATGCGGGCTGCCACCGAGGGCGGCCGCTGGTACCGCGCGCTGGACATCTGGGCCGACATGCGCTCCGCAGGCTGCGAGCCCACCGGCCACGCCTACTCGGCTGCCATCTCGGCCTGCGCCGCCGCAGGCGACTGGCGCCGCGCGGTGGCCCTGTTTGACGAGATGACCGGCCCCGGTGGCATCCGCCCCGACGTGGTCAGCTGCACGGCCCTCATCACGGCGCTGGCGGCTGGCGGCGAGGCGGACCGCGCCGAGGCCGTGGTGGCGTGGATGCTTAGCAACAGCGTGCGGCCCAACGCGCGCACCTACACGGCGCTGATGGCGGCACTGGGCAACGCCAAGCGCTGGGCCCGCGCCGTGGAGGTGCTGGGGCGCATGCAGACCCCCGAGTGGGGCGGCGTGCAGCCCAACGCCTACACGTACTCCGCCTTGCTTAAGGTGGGCCTGAGTTGTTGTGATGATGATGTGTTACAGGGTTGGAAGTGTGCGTTCCTGAACCTGCAACGAGACTAACCGAGCCGCAAGGGCTCCTTGTCTGTGCTGTTAGCGTCATGTTGTGCTCTTTGGAATTAGCCGCGGCTTGTGTCCTGAGTTTGATATGCGCTAGCCCGGGTCCCTTGACCGGTTACTGACTCTGCTGTGCCCTGATCCCCTCCCAATGCAGAGCCTGGGTGAGCACGGCCAGTGGCAGCTGGCTGAGGCCGTGTTCAGCTCTATCGAGCGCCAGGTGCTGGGCCCCGCCGCCGGCTCGGCACCGCCCGCCGCCGCCGCCCTGTCTCTGGCTTCAGCGCTGGTGCCTGCCGCCTCGCCGCCGCCCAGCCCCCTGGCGGCCATGATTGCGGAGGCCAGCGCCACCGCCGCCGCTGCTGCTGCCGCCGTTGCGTCGGCTGCCGGTTCGGCTGCCGTCGCCGCTGCGATCCGCCCGCCTGCCTCGCCCTGCGCGTCTGAATCGTCCTCGTCCGCGCCCTCCCAGTGGACCCGTCCCTCGCAGCTGAACGGACTGTCTCTGGACCTGCACTCCCCCACCTCCACTGGCGGCGCCGTCGAGGCCGGCCCCTCGCGCCGCAGCTTCAGCTTGTTCAGCCACCCGCCTGCGCCTTCGTCCTCCACCGCGACCTCCTGCGCGCCCAGCAGCACCGGCACCAGCGGCCGCTCCTCGTTCAGCGCCGACTCGGCTGTGCCCTCGGACGTCTACTCCGTCGCCAGCACCTCTGCGGCTGAGGCGTGGCGCTCCACTCTGGACCACGCCTCAGCCGCAGCTCTGGCGGGTCAGCTGGCAGCCATGGCGGCGGCCTCGGGCTGCGGCGCCCCCGGCCCCCTGGCCACTGTGCCTGAGGGTGAGCTGGTCACTTTCCCGTCGATCGACGCGGAGGAGGCGTCGACCTCGGCCGCCACCACCCTGGCTCCCGGCGTGTACACCCCCGGCGTGCTGCGTGCCGGTGTGGCTCCGCCTCCCCCTCCTCCCCCTCCGCCCATGTCCTCCTCCGCCCAGCAGCAGCAGCAGCAGCAGTCTGCCTCTACGTCCGGCAACAGCGCCTCCTCGTCCTCCAGCGGCGGTGTGCTGAACGAGGTTGTGTGCGGCGCGCTGATGCTGGCGTACGAGCGTGCCGGCAAGTGGCAGGAGGCCGTGGCGGTGCTGCTGCGGGCGCTGAACCTGGGCATCACCCCCAACACCGTTATGTACAACACCGCTATCTCGGCGGCGGGTAAGGCCGGCCAGCTGGAGATTGCGGAGAAGCTGTACGGCAAGGTGCGGCAGCCGGACGCCGTGACGCACGAGACCATGATTGCGGCGTACGGCATGGCTGGCCTGCCCGAGCGCGCCGAGGCTGTGTTCAGCGCGATGACGTCGGCCGGACTGCGGCCGCGCGACTTCGCCTTCTGCGGCCTCATCGCCGCTCACAGCCTGGCGGGCAACTGGGAGGGCGCTATGCGCGTGCGGGCCCGCATGCGGCGCGCGGGCGTGCAGCCGTCTGTGCATGTGTACAACGCGCTGCTGGCGGCGTGCGAGCGGGCGGGACAGCCCGACCGCGCGCTGGAGCTGTTGGGCGCCATGCGCCGTGAGGGCGTGGAGCCCAACACCCTGACTGCCAACCTGCTGCAGCTGGTGGGTCGCCAGGGCGTGCGCAGCGTGGAGAGCCAGCAGCAGCTGGCGGCCAGTGTCAGTGCAGCGCTGGCGGCCTACGGCGCGCTGCTCATGCAGACCGGTTTGTTCTGA
