# Supplementary material for: Cytosine deaminase as a negative selectable marker for the microalgal chloroplast: a strategy for the isolation of nuclear mutations that affect chloroplast gene expression
Source: Plant J. 2014 Sep 18;80(5):915–25. doi: 10.1111/tpj.12675 (PMC4282525; doi:10.1111/tpj.12675)
Supplement: Supplementary file 6 — Supplementary [file tpj0080-0915-SD6.docx]

**LEGENDS FOR SUPPORTING INFORMATION**

**Cytosine deaminase as a negative selectable marker for the microalgal chloroplast: a strategy for the isolation of nuclear mutations that affect chloroplast gene expression**

Rosanna E. B. Young and Saul Purton

**Figure S1. Analysis of transgenic cytosine deaminase expression in the *Chlamydomonas reinhardtii* chloroplast using non-optimized genes.**

*C. reinhardtii* strain TN72 was transformed with cytosine deaminase genes amplified from three different organisms. The genes were under the control of the *C. reinhardtii atpA* promoter and 5’ UTR and *rbcL* 3’ UTR, and were targeted by homologous recombination into the *psbH* region of the chloroplast. All had a haemagglutinin (HA) tag sequence at the C-terminus. The genes were from the cyanobacterium *Synechocystis* sp. PCC6803 (labelled as ‘Cyano *codA*’; protein accession no. BAA17527), *Escherichia coli* (accession NP_414871) and *Saccharomyces cerevisiae* (labelled as ‘Yeast *codA*’; accession NP_015387).

(a) Western analysis. The blot was probed with an αHA primary antibody and ECL secondary antibody for chemiluminescent detection. Only the *E. coli* CodA enzyme (49 kDa) could be detected. Non-specific bands are present at approximately 25 and 33 kDa in all lanes. CodA protein expression did not correlate with the codon adaptation index (CAI) of the transgenes, calculated with respect to highly-expressed genes in the *C. reinhardtii* chloroplast, but *E. coli codA* does have a higher CAI across the first 45 bp than *codA* from the other two sources. Codon usage near the 5’ end of a gene is thought to be particularly important for translation (Goldman *et al.* 1995).

(b) Demonstration that the natural *E. coli codA* gene (under the *atpA* promoter) does not confer sensitivity to 5-FC upon *C. reinhardtii*.

**Goldman, E., Rosenberg, A.H., Zubay, G., Studier, F.W.** (1995) Consecutive low-usage leucine codons block translation only when near the 5' end of a message in *Escherichia coli.* *J Mol Biol*, **5**, 467-473.

**Figure S2. PCR analysis of *C. reinhardtii* chloroplast transformants, demonstrating correct integration of foreign DNA and homoplasmy.**

**(a)** Agarose gel electrophoresis of PCR products. Template DNA was used from the strains listed above each lane. Three primers were added to each reaction; chloroplast genomes with a parental (TN72) gene layout would lead to an 880 bp product from primers F1 and R1, whereas transformed chloroplast genomes would lead to a 1521/1559 bp product from primers F1 and R2 (left) depending on the size of the promoter/5’ UTR element, or a 1134 bp product from primers F1 and R3 (right). The absence of an 880 bp product in the transformant lanes indicates homoplasmy. Unlabelled lanes contain GeneRuler DNA Ladder Mix (Thermo Scientific).

**(b)** Representation of primer binding positions on the recipient and transformed chloroplast genomes.

**Table S1. UV mutants of *C. reinhardtii* cell line A1 can be complemented with the *TAA1* plasmid.**

Selection was carried out on HSM (minimal medium) in the light; integration of the *TAA1* plasmid into the nuclear genome restores phototrophic growth in all four of the cell lines tested.

**Table S2. Primers used for the identification of mutations in nuclear genes.**

Genes were amplified from *C. reinhardtii* genomic DNA in sections using Phusion Polymerase, GC buffer and 3% DMSO (all Thermo Scientific). 320 ng template DNA was used per 25 μl reaction. Products were cleaned using a PCR Purification Kit (Thermo Scientific) before DNA sequencing.

**APPENDIX S1. DNA and translated sequences used in this work.**
